# SOCS-1 rescues IL-1β-mediated suppression of epithelial sodium channel in mouse lung epithelial cells via ASK-1

**DOI:** 10.18632/oncotarget.8543

**Published:** 2016-04-01

**Authors:** Lakshmi Galam, Ramani Soundararajan, Mason Breitzig, Ashna Rajan, Rajashekar Reddy Yeruva, Alexander Czachor, Francine Harris, Richard F Lockey, Narasaiah Kolliputi

**Affiliations:** ^1^ Division of Allergy and Immunology, Department of Internal Medicine, Morsani College of Medicine, University of South Florida, Tampa, FL 33612, USA

**Keywords:** SOCS-1, edema, lung injury, inflammation, ASK-1

## Abstract

**Background:**

Acute lung injury (ALI) is characterized by alveolar damage, increased levels of pro-inflammatory cytokines and impaired alveolar fluid clearance. Recently, we showed that the deletion of Apoptosis signal-regulating kinase 1 (ASK1) protects against hyperoxia-induced acute lung injury (HALI) by suppressing IL-1β and TNF-α. Previously, our data revealed that the suppressor of cytokine signaling-1 (SOCS-1) overexpression restores alveolar fluid clearance in HALI by inhibiting ASK-1 and suppressing IL-1β levels. Furthermore, IL-1β is known to inhibit the expression of epithelial sodium channel α-subunit (ENaC) via a p38 MAPK signaling pathway.

**Objective:**

To determine whether SOCS-1 overexpression in MLE-12 cells would protect against IL-1β-mediated depletion of αENaC by suppressing ASK-1 expression.

**Methods:**

We co-transfected MLE-12 cells with SOCS-1 overexpressing plasmid with or without IL-1β in the presence or absence of sodium channel inhibitor, amiloride. We measured potential difference, transepithelial current, resistance, and sodium uptake levels across MLE-12 cells. We studied the effect of ASK-1 depletion, as well as ASK-1 and SOCS-1 overexpression on αENaC expression.

**Results:**

SOCS-1 overexpression sufficiently restored transepithelial current and resistance in MLE-12 cells treated with either IL-1β or amiloride. The αENaC mRNA levels and sodium transport were increased in SOCS-1 overexpressing MLE-12 cells exposed to IL-1β. Depletion of ASK-1 in MLE-12 cells increased αENaC mRNA levels. Interestingly, SOCS-1 overexpression restored αENaC expression in MLE-12 cells in the presence of ASK-1 overexpression.

**Conclusion:**

Collectively, these findings suggest that SOCS-1 may exert its protective effect by rescuing αENaC expression via suppression of ASK-1.

## INTRODUCTION

Acute lung injury (ALI) is a debilitating syndrome characterized by alveolar barrier damage in alveolar capillary endothelial cells and type I pneumocytes, resulting in a large uptake of fluid and macromolecules [1]. In its acute phase, ALI results in respiratory failure with a mortality rate of 30-40%, which is in part attributed to compromised cell permeability. The most common cause of ALI in patients is sepsis, pneumonia, or surgical procedures that involve transfusions and gastric aspirations. Patients subjected to such conditions exhibit activation of inflammatory mediators, cytokines (e.g., IL-1β, IL-8, and TNFα), and lipid mediators (e.g. LT B_4_) in lung tissue [2]. While a number of inflammatory mediators are upregulated during the disease state, IL-1β remains the most biologically active cytokine that augments alveolar epithelial repair and is a critical initiator of fibroblast activation and proliferation [3, 4]. In ALI, along with elevation of inflammatory mediators [5] and aberrant surfactant production [6], there is a distinct influx of protein-rich edema fluid into the interstitial and alveolar spaces, leading to impaired alveolar fluid clearance in ALI patients [7]. IL-1β is found in the pulmonary edema and bronchioalveolar lavage (BAL) fluids of ALI patients [5, 8, 9]. To mimic the ALI pathology *in vivo*, hyperoxia-induced mouse models have been used. Hyperoxia-induced oxidative stress is a critical model that is indicative of the toxic effects produced by acute lung injury, such as impairing the viability of epithelial and endothelial barriers, inhibiting oxidative phosphorylation, and inducing the release of toxic aldehydes (e.g. 4-HNE, MDA) and pro-inflammatory cytokines [10, 11]. Previously, we have shown that hyperoxia damages mitochondrial morphology and induces the release of pro-apoptotic factors, such as caspase-1, and pro-inflammatory cytokines such as IL-1β, revealing physiological reactions observed in ALI [12]. However, the factors that lead to reduced alveolar clearance in ALI have not been well studied. The removal of lung edema fluid from airspaces occurs through an active sodium transport gradient across the distal lung epithelium [3, 13].

The Epithelial sodium channel (ENaC) is composed of α, β, and γ subunits. Sodium transport via αENaC expression in alveolar epithelial cells acts as the driving force for fluid removal from the alveolar space to facilitate physiological gas exchange and for sodium ion-mediated transepithelial reabsorption [14–17]. However, under inflammatory conditions, IL-1β suppresses αENaC expression and affects the alveolar epithelial sodium transport, where there is a vast restriction of water and ion transport across distal lung epithelium. Mechanistically, IL-1β decreases αENaC mRNA expression by inhibiting its promoter activity via a p38 mitogen-activated protein kinase (MAPK) dependent signaling pathway [18]. However, the mechanism(s) involved in mediating the downstream effects of IL-1β on p38 MAPK pathway and rescuing αENaC transcription remain unclear. Several other known underlying mechanisms exist, in addition to IL-1β, which may contribute to the loss of αENaC expression and activity. TNFα, a well studied inflammatory cytokine, has been identified to decrease αENaC activity via transcriptional suppression [19]. Furthermore, TGF-β has similarly been identified to inhibit αENaC activity through direct binding, internalizing αENaC from the alveolar epithelial cell surface [20].

Interestingly, an upstream component of p38 MAPK pathway is an Apoptosis signal–regulating kinase-1 (ASK-1). Reactive oxygen species (ROS), and certain proinflammatory cytokines such as TNF-α activate ASK-1, which in turn activates the p38 MAPK pathway, initiating cell death [21]. SOCS-1 is known to exert its protective effects against apoptosis initiated by cytokines TNF-α and INF-γ [22–24]. Our recent report indicates that the SOCS-1 expression is associated with IL-6 mediated cytoprotection against hyperoxic acute lung injury (HALI) and the mechanism involves SOCS-1- induced ASK-1 degradation [25]. In addition, our study reveals that the overexpression of SOCS-1 is protective against hyperoxic lung injury via decreased ASK-1 expression [26].

In this study, we report for the first time that SOCS-1 overexpression rescues IL-1β- induced suppression of αENaC expression in mouse lung epithelial (MLE-12) cells. Furthermore, we show that SOCS-1-induced αENaC expression, as well as the restoration of sodium transport across mouse lung epithelial cells, involves the suppression of ASK-1.

## RESULTS

### SOCS-1 suppresses the effect of IL-1β on PD and TEC across MLE-12 cells

**Figure 1:**
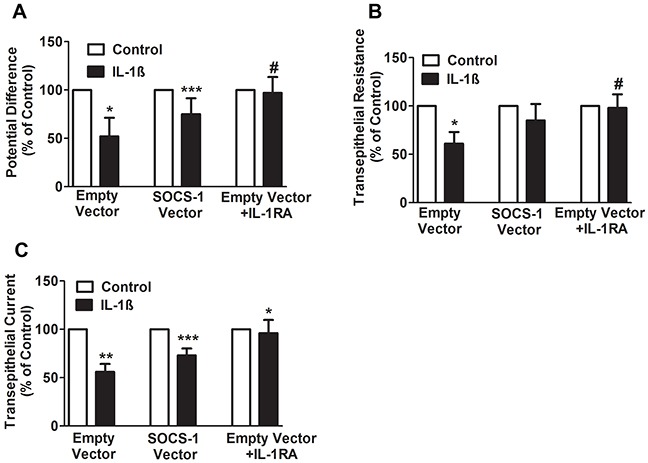
SOCS-1 rescues the IL-1β mediated decrease in PD, TER, and TEC **A-C.** MLE cells were transfected with control or SOCS-1 vector in the presence or absence of IL-1β (10ng/ml) or IL-RA (10 μg/ml). The PD, TER, and TEC were measured with Millicell-ERS Voltohmmeter. Results are represented as means ± SEM, n = 4-5 per group). Each experiment was performed thrice. The results are expressed as percentage of control. **A.** For PD, * P< 0.05 relative to empty vector, *** P< 0.001 versus empty vector and # = * P< 0.05 relative to empty vector. **B.** For TER measurements, * P < 0.05 relative to empty vector and # = * P < 0.05 versus empty vector. **C.** For TEC, ** P < 0.01 versus empty vector and *** P < 0.001 relative to empty vector. * P < 0.01 versus SOCS-1 vector.

Our recent study indicates that the adenoviral transfer of SOCS-1 is associated with increased alveolar fluid clearance in mice exposed to hyperoxia [26], which is a clinically relevant model used to study acute lung injury in murine models [10, 27]. In this study, to determine the mechanism of SOCS-1 protection against hyperoxic lung injury, MLE-12 cells were transfected with either empty vector or SOCS-1, treated with IL-1β followed by measurement of potential difference (PD), trans-epithelial resistance (TER) and trans-epithelial current (TEC). In MLE-12 cells transfected with empty vector and treated with IL-1β, we observed a significant two-fold decrease in PD, relative to the untreated control group (Figure 1A). Interestingly, MLE-12 cells transfected with SOCS-1 demonstrated a significant increase in PD when subjected to IL-1β treatment (Figure 1A). In the presence of IL-1 receptor antagonist (IL-1RA), the protective effect of SOCS-1 on PD was reversed (Figure 1A). Similarly, we observed a significant two-fold decrease in TER in IL-1β-treated MLE-12 cells transfected with empty vector and this effect that was blocked by IL-1RA (Figure 1B). However, SOCS-1 overexpression in MLE-12 cells treated with IL-1β did not change TER. (Figure 1B). Furthermore, IL-1β treated MLE-12 cells expressing empty vector showed a significant decrease in the calculated TEC (Figure 1C). Overexpression of SOCS-1 in MLE-12 cells treated with IL-1β caused an increase in TEC (Figure 1C). This protective effect of SOCS-1 was abrogated in the presence of IL-1RA (Figure 1C). Collectively, the data suggests a protective role of SOCS-1 in MLE-12 exposed to IL-1β.

### SOCS-1 has suppressive effect on IL-1β on both apical and basal surfaces of MLE-12 cells

**Figure 2:**
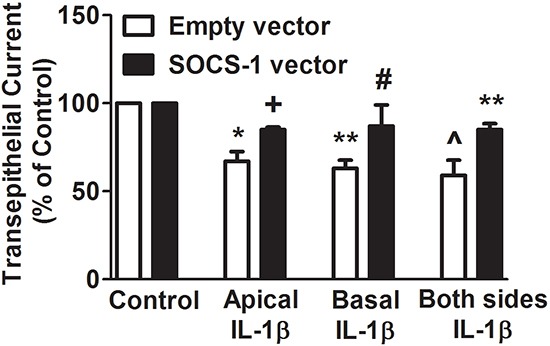
SOCS-1 overexpression suppresses IL-1β-mediated disruption of TEC on apical, basal as well as both surfaces of MLE-12 cells To determine whether SOCS-1 has a greater attenuating effect on IL-1β damage on the apical or basal or both surfaces, MLE-12 cells were treated in the presence or absence of IL-1β (10 ng/ml) in the apical or basal or both surfaces with or without SOCS-1 overexepression. TEC was measured using the Millicell-ERS Voltohmmeter, calculated using Ohm's Law as described in the Methods. TEC is represented as means ± SEM, n = 4-5 per group). Each experiment was performed thrice. TEC is expressed as percentage of control. * P < 0.05, ** P < 0.01 compared to empty vector. ^ = * P < 0.05 versus empty vector. + = *P < 0.05 compared to empty vector treated with IL-1β on apical surface. # = * P < 0.05 versus empty vector treated with IL-1β on basal surface. ** P < 0.01 compared to empty vector treated with IL-1β on both surfaces.

Previous studies indicate that the effect of IL-1β on several epithelial cell lines including distal colon, renal collecting duct and normal human bronchial epithelial cells is primarily on the apical surface of the cells [28, 29]. To determine whether SOCS-1 displays a preferential suppression of IL-1β in apical or basal or both surfaces the TEC was analyzed in MLE-12 cells that were treated with IL-1β on the aforementioned surfaces with or without SOCS-1. In MLE-12 cells treated with either empty or SOCS-1 vector, there was no change in TEC in the absence of IL-1β (Figure 2). When MLE-12 cells overexpressing SOCS-1 were exposed to IL-1β on their apical surface, there was a marginal increase in the TEC when compared to the empty vector controls (Figure 2). Similar results were obtained when either the basal or apical or both surfaces of MLE-12 cells were exposed to IL-1β (Figure 2). Collectively, our data correlates with several previous studies [18] which suggest that the cytokine IL-1β increases paracellular permeability, thus enabling it to diffuse across the monolayer, contributing to lack of sidedness. Therefore, its action is inhibited by SOCS-1 on both sides (Figure 2).

### SOCS-1 suppresses the effect of IL-1β on the sodium transport across mouse lung epithelial cell monolayers

**Figure 3:**
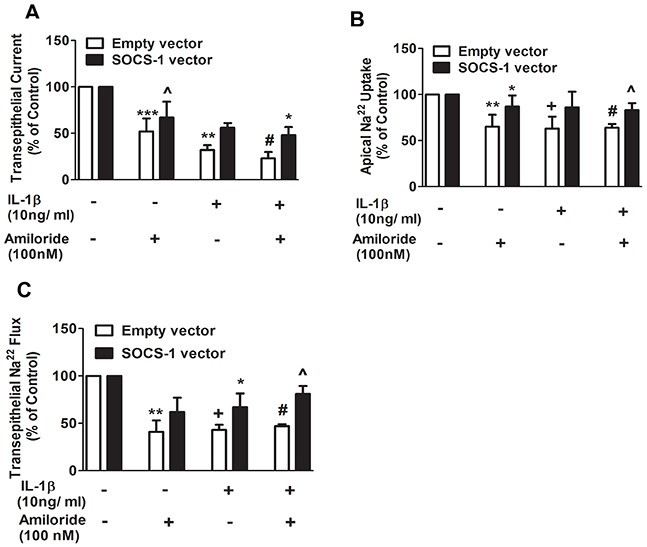
Apical ^22^Na and transepithelial ^22^Na flux are proteced by SOCS-1 overexpression **A-C.** To assess the potential protective effect of SOCS-1 on sodium transport, across MLE-12 cells, were transfected with empty or SOCS-1 vector and treated with amiloride (100 nM) or IL-1β (10 ng/ml), or both. TEC was measured using Millicell-ERS Voltohmmeter. Both apical ^22^Na and transepithelial ^22^Na flux were analyzed using a β-counter to measure radioactivity in the cell lysates as described in Methods. Results are represented as means ± SEM, n = 4-5 per group). Each experiment was performed thrice. The results are expressed as percentage of control. (A) *** P < 0.001, ** P < 0.01 and + = *** P < 0.001 versus empty vector. # = ** P < 0.01 versus MLE cells expressing empty vector and treated with amiloride. * P < 0.05 relative to MLE-12 cells expressing empty vector and treated with amiloride and IL-1β. (B) ** P < 0.01, + = ** P < 0.01 and # = ** P < 0.01 relative to empty vector alone. * P < 0.05 versus amiloride treated MLE-12 cells expressing empty vector. ^ = * P < 0.05 relative to amiloride and IL-1β treated MLE-12 cells expressing empty vector. (C) ** P < 0.01, + = ** P < 0.01 and # = ** P < 0.01 versus empty vector. * P < 0.05 versus MLE-12 cells expressing empty vector and treated with IL-1β. ^ = * P < 0.05 relative to MLE-12 cells expressing empty vector and treated with IL-1β and amiloride.

Previously, it has been reported that IL-1β decreases αENaC expression in alveolar type II (ATII) cells and inhibits alveolar sodium transport [18]. αENaC expression and TEC are positively correlated [30]. Because SOCS-1 overexpression causes an increase in TEC, we sought to determine the effect of SOCS-1 on sodium transport. We subjected MLE-12 cells transfected with either empty vector (control) or SOCS-1 vector, to amiloride (100nM) or IL-1β (10ng/ml) respectively, or a combination of both amiloride (100nM) and IL-1β (10 ng/ml). The TEC, transepithelial ^22^Na flux and apical ^22^Na uptake were then measured in each case (Figure 3A–3C). When MLE-12 cells were exposed to amiloride, an inhibitor of αENaC, there was a significant decrease in TEC relative to MLE-12 cells transfected with either empty or SOCS-1 vector (Figure 3A). However, SOCS-1 overexpressing cells treated with amiloride showed a significant increase in TEC compared to amiloride-treated controls (Figure 3A). The presence of IL-1β had the same effect on the two groups of cells with SOCS-1 vector, exhibiting a suppressive effect on the IL-1β-induced decrease in TEC (Figure 3A). A synergetic effect of amiloride and IL-1β treatment was observed in control cells with a significant 5-fold decrease in TEC (Figure 3A). This substantial decrease in TEC was counteracted by MLE-12 cells overexpressing SOCS-1. Similarly, there was a significant decrease in apical ^22^Na uptake upon amiloride treatment and this was rescued by SOCS-1 overexpression (Figure 3B). A similar result was obtained upon IL-1β treatment in these two groups (Figure 3B). Interestingly, SOCS-1 overexpression led to an increase in apical ^22^Na uptake by MLE-12 cells in the presence of both IL-1β and amiloride (Figure 3B). Next, we evaluated the transepithelial ^22^Na uptake in MLE cells. There was a significant decrease in transepithelial ^22^Na uptake in amiloride treated MLE-12 cells (Figure 3C). Similar results were obtained with IL-1β treatment or combined treatment of amiloride and IL-1β in MLE-12 cells (Figure 3C). MLE-12 cells overexpressing SOCS-1 rescued the effect of amiloride or IL-1β, or a combination of both, by significantly increasing the transepithelial ^22^Na uptake in MLE-12 cells. Altogether, this data suggests that SOCS-1 unequivocally suppresses the deleterious effect of IL-1β on MLE-12 cells, specifically on amiloride-sensitive ^22^Na transport channels, thus establishing a protective influence in ALI.

### Suppression of αENaC gene expression by IL-1β is counteracted by SOCS-1 through inhibition of ASK-1 in MLE-12 cells

**Figure 4:**
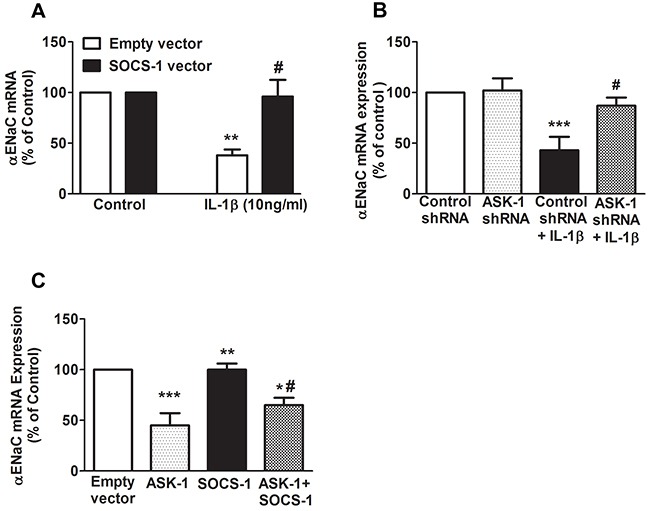
SOCS-1 rescues IL-1β mediated decrease of αENaC mRNA expression by ASK-1-dependent pathway We carried out transfection studies in MLE-12 cells to study the effects of SOCS-1 or ASK-1 overexpression or ASK-1 depletion on αENaC mRNA expression. **A.** MLE-12 cells were transfected with SOCS-1 or empty vector and treated with or without IL-1β (10 ng/ml) for 36 hours. Total RNA was extracted and αENaC expression was analyzed by real-time RT-PCR. **B.** MLE-12 cells were transfected with either control or ASK-1 shRNA for 36 hours and treated in the presence or absence of IL-1β (10 ng/ml) for 36 hours. Total RNA was extracted and αENaC expression checked using real-time PCR. **C.** MLE-12 cells were transfected with ASK-1 or SOCS-1 vector for 36 hours and again analyzed for αENaC expression using the above method. Results are represented as means ± SEM, N = 4-5 per group. Each experiment was performed thrice. The results are expressed as percentage of control. (A) ** P < 0.01 versus empty vector. # = * P < 0.05 versus MLE-12 cells expressing empty vector and treated with IL-1β. (B) *** P < 0.001 relative to control shRNA. # = ** P < 0.01 versus control shRNA treated with IL-1β. (C) *** P < 0.001 versus empty vector. ** P < 0.01 versus ASK-1 overexpressing MLE-12 cells. * P < 0.05 versus SOCS-1 expressing MLE-12 cells. # = * P < 0.05 compared to ASK-1 overexpressing MLE-12 cells.

Previously, it has been demonstrated that IL-1β decreases αENaC transcription through p38 MAPK phosphorylation of αENaC promoter [18]. To further determine whether SOCS-1 mediated rescue of IL-1β leads to a decrease in sodium transport and to identify if this rescue has any effect on αENaC mRNA expression, MLE-12 cells were transfected with empty vector or SOCS-1 vector. Our results shows that MLE-12 cells overexpressing SOCS-1 showed no significant difference in αENaC mRNA expression when compared to MLE-12 cells expressing empty vector (Figure 4A). However, when MLE-12 cells expressing empty vector were treated with IL-1β, there was a significant decrease in αENaC mRNA expression (Figure 4A). This antagonistic effect of IL-1β on αENaC mRNA was reversed by SOCS-1 overexpression (Figure 4A).

Previously, we have shown that SOCS-1 overexpression in mouse lungs confers protection in hyperoxic lung injury via inhibition of hyperoxic-induced ASK-1 expression [26]. To further determine if SOCS-1 regulation of ASK-1 has any effect on αENaC mRNA expression, MLE-12 cells were transfected with either control shRNA or ASK-1 shRNA (Figure 4B). Our data shows that shRNA mediated knockdown of ASK-1 mRNA in MLE-12 cells displayed no change in αENaC transcript relative to cells treated with control shRNA (Figure 4B). In the presence of IL-1β, control cells showed a significant decrease in αENaC mRNA levels, whereas in cells wherein ASK-1 transcript level was abrogated, there was a significant increase in αENaC mRNA levels (Figure 4B). Additionally, we overexpressed ASK1 or SOC-1 or both ASK-1 and SOCS-1 in MLE-12 cells and evaluated its effect on αENaC mRNA expression. We observed that ASK-1 overexpression caused a significant decrease in αENaC mRNA expression in MLE-12 cells (Figure 4C). However, there was no change in αENaC mRNA expression in SOCS-1 overexpressing MLE-12 cells (Figure 4C). Overexpression of both ASK-1 and SOCS-1 in MLE-12 cells restored the αENaC mRNA levels relative to ASK-1 overexpression alone (Figure 4C).

## DISCUSSION

ALI is a devastating syndrome characterized by damage to alveolar membrane permeability and disruption of sodium uptake [31]. Earlier studies have shown that IL-1β is one of the most biologically active cytokines found in the airspace of ALI patients [5, 8, 9]. IL-1β has been shown to cause an increase in protein permeability across the lung alveolar capillary barrier [32], inhibit fluid transport in distal lung epithelium [18] and cause surfactant abnormalities [33]. In addition, IL-1β has been implicated in inhibiting alveolar epithelial sodium transport across the distal lung epithelium via a p38 MAPK-dependent pathway involving reduced αENaC expression [18]. A recent study from our laboratory has shown that the overexpression of SOCS-1 is protective against hyperoxic lung injury via the inhibition of ASK-1 expression [26]. However, the role of SOCS-1 in reversing the IL-1β mediated decrease in αENaC expression and inhibition of alveolar epithelium transport across distal epithelium remains unknown. In addition, the effect of ASK-1 overexpression on αENaC mRNA transcript has not been explored. These questions will be further addressed in the present study.

We found that IL-1β significantly decreased transepithelial PD, TER and TEC in MLE-12 cells and that its effects were reversed by the use of IL-1RA or the overexpression of SOCS-1. This identifies a strong correlation between SOCS-1 and the IL-1β-mediated electrophysiological characteristics. The effect of IL-1β on MLE-12 cells is in agreement with a recent study wherein alveolar type II monolayers treated with IL-1β exhibited a decrease in TEC in a time and dose-dependent manner that was blocked by IL-1RA treatment [18]. SOCS-1 attenuated IL-1β-mediated cellular damage independent of application of IL-1β on apical, basal, or both surfaces of MLE-12 cells. This correlates well with a report where a lack of sidedness of IL-1β effect was linked to the transient nature of IL-1β-mediated increase in cellular permeability [18] and contrasts with a TGF-β-mediated decrease in TEC in alveolar type II monolayers predominantly on the basolateral side [34]. This suggests that the protective effect of SOCS-1 overexpression that we observed in our previous study [26] may be due attenuation of IL-1β-mediated αENaC suppression. Therefore, this study has given us insight as to how SOCS-1 enhances alveolar fluid clearance. [26]. SOCS-1 is also understood to actively play a role in targeting proteins for polyubiquitination, leading to their degradation via the proteosomal pathway [35]. In addition, similar studies have indicated that the expression of Nedd4-2, an E3 ubiquitin ligase is responsible for the proteosomal degradation of αENaC [36], therefore SOCS-1 may influence Nedd4-2. Future studies are required in order to better understand the link between SOCS-1 and Nedd4-2.

Earlier experiments show that SOCS-1 restores majority of IL-1β-mediated cellular damage during hyperoxic lung injury [25]. Moreover, the anti-inflammatory and anti-apoptotic effect of SOCS-1 have been well documented in several studies [25, 37, 38]. In our study, overexpression of SOCS-1 in MLE-12 cells rescued TEC suggesting it may play a role in restoring the sodium transport across MLE-12 monolayers that was decreased upon IL-1β treatment. Here, we report for the first time the protective effect of SOCS-1 overexpression on amiloride-sensitive sodium transport and TEC across MLE-12 cells. The disruption of amiloride-sensitive sodium transport by IL-1β in our study is in concordance with other studies where IL-1β has been shown to inhibit apical sodium transport in distal colon and the renal collecting duct, as well as type II alveolar epithelial cells [18, 28, 29]. Interestingly, similar to our previous findings, these results confirm that normal bronchial epithelial cells treated with IL-1β showed a decrease in the amiloride-sensitive short current [39].

One of the most important sodium channels in lung epithelial cells is αENaC, which is required for fluid and salt absorption. It functions by keeping the lung airspace dry, thus facilitating gas exchange [15–17, 40]. We found that, in concordance with a previous report [18], IL-1β similarly decreased αENaC transcript levels in MLE-12 cells. Moreover, as SOCS-1 overexpression restored sodium uptake, trans-epithelial ^22^Na flux and TEC in MLE-12 cells, we hypothesized that SOCS-1 may play an influential role in mediating regulators of transepithelial ion transport. Our results indicate that SOCS-1 overexpression was sufficient for restoration of αENaC expression even in the presence 1L-1β.

This brings us to the most fundamental question: how does 1L-1β affect the sodium transport? Secondly, how does SOCS-1 signaling induce protection against IL-1β mediated cellular damage? In a study conducted by Roux *et. al*. (2005), IL-1β was shown to decrease αENaC mRNA via activation of p38 MAPK pathway. Also, active TGF- β1 was shown to decrease both αENaC gene expression and protein levels in rat and human ATII cells [34]. In another related study, IL-1β mediated protein permeability across alveolar epithelial cell monolayers was shown to bedue to transforming growth factor activation via RhoA/αvB6 integrin-dependent mechanisms [41]. Previously, we have shown that SOCS-1 is an upstream regulator of the ASK-1-JNK pathway in hyperoxic lung injury model [25]. Additionally, we have recently established the cytoprotective role of SOCS-1 overexpression in hyperoxic lung injury via ASK-1 depletion [26]. It is well documented that ASK-1 is upstream of p38 MAPK pathway [21]. Therefore, in the present study, we further explored the SOCS-1-ASK1 signalling pathway in relation to αENaC expression. We identify that overexpression of ASK-1 has led to a decrease in αENaC expression. Interestingly, SOCS-1 overexpression attenuated the deleterious effect of ASK-1 on αENaC expression. Interestingly, previous studies of ours identified ASK-1 deletion to result in the protection against ALI [42]. Taken together with current findings in the present study, which identifies ASK-1 overexpression to result in αENaC mRNA suppression, ASK-1 may be involved in the pathophysiology of ALI. This further implicates ASK-1 signaling in SOCS-1 mediated protection against deleterious effect of IL-1β. In summary, the results of this study reveal that SOCS-1 is sufficient to ameliorate the effects of IL-1β by depleting levels of ASK-1. Due to the failure of alveolar epithelial cells to disseminate fluids, which leads to further cellular damage and the progression of ALI, we surmise that SOCS-1 may be a viable target for remediation of vectorial fluid clearance in ALI.

## MATERIALS AND METHODS

### Reagents

Recombinant human IL-1β and IL-1RA were obtained from R&D Systems (Minneapolis, MN). ^22^Na was obtained from Perkin Elmer Life. Amiloride and all other reagents were purchased from Sigma (St. Louis, MO). All cell culture media were prepared by the University of South Florida Cell Culture Facility using deionized water and analytical grade reagents.

### Cell culture

Mouse lung epithelial cells (MLE-12) were used for *in-vitro* studies (ATCC, Manassas, VA). The culture medium was supplemented with growth factors and antibiotics according to the manufacturer's instructions [43]. Confluent cultured cells were treated with IL-1β every 3 hours at 37°C, and then the medium was removed and replaced with standard growth medium as previously described [43]. Twenty-four hours later, PBS was used to clear non-adherent epithelial cells and fresh medium was added. After 72–96 hours, cells that formed confluent monolayers and developed a TER (1500 Ohms.cm^2^) were used for further experiments.

### Plasmid constructs

We received mammalian expression plasmid for wild-type (WT) ASK-1 from Dr. Wang Min of Yale University as described previously [44]. The wild-type SOCS-1 expression plasmid used in this study was given by Dr. Tadamitsu Kishimoto [38] from Osaka University, Japan.

### Transfection

For transfection studies, we transfected MLE-12 cells with either control shRNA or ASK-1 shRNA for 36 hours using Lipofectamine 2000-plus as per manufacturer's protocol (Invitrogen, Carlsbad, CA). Similarly, we transfected MLE-12 cells with plasmid overexpressing SOCS-1 for 36 hours using Lipofectamine 200-plus as described previously [25]. Briefly, we seeded a confluent culture (90%) of MLE-12 cells in six-well plates and then transfected cells with 4 μg of plasmid DNA. The medium was changed every 12 hours after post-transfection. The non-targeted β-Gal shRNA used as a control (sense sequence, UUAUGCCGAUCGCGUCACAUU) was obtained from Santa Cruz and ASK-1 shRNA (catalog number sc-29748) was obtained from Santa Cruz Biotechnology (Santa Cruz, CA). MLE-12 cells were transfected with either control or ASK-1 shRNA using DharmaFECT following the manufacturer's protocol (Dharmacon, Lafayette, CO). 36–48 hours post-transfection, cells were harvested and the prepared cell lysates were then used for protein estimation (Biorad reagent).

### Measurement of transepithelial PD, TER and TEC

MLE-12 cells were transfected with control shRNA or ASK-1 shRNA with or without SOCS-1 vector for 36 hours in the presence or absence of amiloride (100 nM). IL-1β (10 ng/ml) was added on the apical or basal or both surfaces of the cell monolayer before measurements were made. Following treatment, TER kOhms.cm^2^ and potential difference (PD;mV) were analyzed using the Millicell-ERS Voltohmmeter (Millipore Corp., Bedford, MA) with Ag/AgCl electrodes, as described previously [45]. TEC (μA/cm^2^) was calculated from Ohm's Law equation: TEC = PD/R_t_, where R_t_ is the resistance. The effect of IL-1β (10 ng/ml for 1–24 hours) or its control on the bioelectric properties of MLE-12 cells was evaluated on day 4 in culture. The data are represented as percentage of control.

### Measurement of sodium uptake

Sodium transport across MLE-12 cells was evaluated by unidirectional tracer uptake measurements using the technique that was previously described [46]. Briefly, after exposure of cells to IL-1β (10 ng/ml) or vehicle, the cells were washed twice with PBS (150 mM NaCl and 2 mM HEPES, pH 7.4) at 37°C and equilibrated with flux medium (140 mM NaCl, 5 mM KCl, 1 mM Na_2_HPO4, 1 mM MgCl_2_, 0.2 mM CaCl_2_, 10 mM glucose, and 20 mM HEPES, pH 7.4) for 10 minutes at 37°C. After equilibration, the medium containing 5 μCi/ml ^22^Na and ouabain (3 mM) was added to the cells. After 6 min incubation, cells were washed three times with cold PBS to clear excess of Na^22^ and halt the uptake by cells. The final rinse was verified for absence of ^22^Na in the medium. Following these treatments, the cells were lysed using 0.1% NaOH, and radioactivity was measured using a β-counter. The results were normalized by protein estimation.

### Measurement of transepithelial sodium flux

To measure transepithelial sodium flux, the activity of the amiloride-sensitive sodium transport across MLE-12 cell monolayers was determined by unidirectional tracer transport measurements, a technique adapted from Mairbaurl *et al*., 1997 [46]. Briefly, MLE-12 cells were treated with IL-1β or vehicle and washed twice with PBS at 37°C and equilibrated with flux medium for 10 minutes at 37°C as described previously. After equilibration, cells were treated with fresh flux medium containing ^22^Na at a final concentration of 5 μCi/ml. After 10 minutes, the radioactivity was measured in the medium by using a β-counter. The cells were then lysed with 0.1% NaOH, and the protein was estimated for normalization of the results.

### Quantitative Real-time RT-PCR

Total RNA was extracted from MLE-12 cells using RNeasy kit as per manufacturer's instructions (Qiagen, Valencia, CA). Total RNA was reverse transcribed using iScript cDNA synthesis kit according to the manufacturer's recommendations (BioRad Laboratories, Hercules, CA). Quantitative RT-PCR was performed using SYBR green supermix, specific primers and cDNA in a BioRad iCycler (BioRad Laboratories, Hercules, CA) as per manufacturer's instructions. qRT-PCRs were performed using a 2-step amplification protocol as follows: initial denaturation at 95°C for 3 minutes followed by 40 cycles at 95°C for 15 seconds (denaturation) and 60°C (amplification and extension) for 30 seconds. The αENaC transcript level was measured using αENaC RT^2^qPCR Assay primers. The 18S rRNA was used as an internal calibrator. The relative fold change in αENaC transcript was determined by ΔΔ*C*_T_ method [47]. The results are expressed as percentage of control and were used for statistical analyses.

### Statistical analysis

For statistical analysis, individual group means were compared with Student's unpaired *t* test. For larger datasets involving more than two groups, one-way analysis of variance (ANOVA) with post-hoc Tukey test was used. P-value < 0.05 was considered to be significant.
